# Successful Control of an Onboard COVID-19 Outbreak Using the Cruise Ship as a Quarantine Facility, Western Australia, Australia

**DOI:** 10.3201/eid2705.204142

**Published:** 2021-05

**Authors:** Tudor A. Codreanu, Sera Ngeh, Abigail Trewin, Paul K. Armstrong

**Affiliations:** Department of Health Western Australia, Perth, Western Australia, Australia (T.A. Codreanu, S. Ngeh, P.K. Armstrong);; National Critical Care and Trauma Response Centre, Darwin, Northern Territory, Australia (A. Trewin)

**Keywords:** Western Australia, disease outbreaks, COVID-19, quarantine, cruise ships, coronavirus disease, SARS-CoV-2, severe acute respiratory syndrome coronavirus 2, viruses, respiratory infections, zoonoses, Australia

## Abstract

Onboard quarantining has been only partially effective to control outbreaks of coronavirus disease on cruise ships. We describe the successful use of the ship as a quarantine facility during the response to the outbreak on the MS Artania, which docked in Western Australia, Australia. The health-led 14-day quarantine regime was based on established principles of outbreak management and experiences of coronavirus disease outbreaks on cruise ships elsewhere. The attack rate in the crew was 3.3% (28/832) before quarantine commencement and 4.8% (21/441) during quarantine on board. No crew members became symptomatic after completion of quarantine. Infection surveillance involved telephone correspondence, face-to-face visits, and testing for severe acute respiratory syndrome coronavirus 2. No serious health issues were reported, no response staff became infected, and only 1 quarantine breach occurred among crew. Onboard quarantine could offer financial and operational advantages in outbreak response and provide reassurance to the shore-based wider community regarding risk for infection.

Cruise ships are a highly susceptible environment for the rapid spread of infectious diseases because of high population density, encouragement of social interaction, and common food and water sources. A variety of pathogens have been implicated, including norovirus (*1*), influenza virus (*2*), *Legionella pneumophila* (*3*), *Cyclospora* (*4*), *Salmonella enterica* serotype Enteriditis (*5*), and measles (*6*). Passengers are often elderly and have underlying conditions that put them at higher risk for health complications after infection.

The use of quarantine (i.e., “the restriction of activities of or the separation of persons who are not ill but who may have been exposed to an infectious agent or disease” [*7*]) has been a cornerstone of infectious disease control for centuries. Quarantine ensures the early detection of cases by monitoring for illness onset and isolating infected persons from others until they are no longer infectious (*8*). In modern times, the period of quarantine is normally set at the maximum incubation period of the disease of interest (*9*). Quarantine has been used, in conjunction with other measures, to control infectious disease outbreaks on cruise ships (*10*–*12*).

On January 7, 2020, severe acute respiratory syndrome coronavirus 2 (SARS-CoV-2) was identified as the causative organism of an infectious respiratory disease affecting residents of Wuhan, China. Coronavirus disease (COVID-19) rapidly spread around the world and was declared a pandemic on March 12, 2020 (*13*). Outbreaks of COVID-19 on cruise ships were an early feature of the pandemic, and quarantine was used to varying degrees as a control measure. One of the earliest and largest outbreaks of COVID-19 on a cruise ship was reported aboard the Diamond Princess, which arrived in Yokohama, Japan, on February 3, 2020; ultimately, 712 of 3,711 (19.2%) passengers and crew contracted the infection, and 13 persons died (*14*). On February 5, the government of Japan instituted a 14-day quarantine period on board the Diamond Princess (*15*,*16*). Quarantined passengers were allowed periods outside their cabins for health and well-being, and crew continued their usual duties after quarantine began (*14*,*15*,*17*). SARS-CoV-2 continued to be transmitted on board within passenger cabins and by infected food service workers (*15*,*17*–*19*). This quarantine measure proved effective in decreasing transmission; however, it did not completely control the outbreak, and further cases occurred after release from quarantine (*14*,*17*–*20*).

The cruise ship MS Artania, which is 230 meters long, 9 decks, and built in 1984, can carry <1,260 passengers in 594 cabins and 537 crew members in 321 cabins. Departing Hamburg, Germany, on December 21, 2019, for a 6-month world tour, the ship arrived in Fremantle Port, Western Australia (WA), on March 25, 2020, carrying 832 passengers (age range 7–89 years) of 12 nationalities and 503 crew members (age range 23–61 years) of 30 nationalities.

On arrival, the ship’s medical team reported to WA health authorities that 2 passengers had tested positive for SARS-CoV-2 upon their return to Germany after disembarking the ship in Sydney, Australia, on March 14, 2020, and that a further 15 passengers and 10 crew had reported fever, mild respiratory symptoms, or both during March 21–25. Point-of-care test kits for influenza A and B were not available on board. According to standard ship protocols, these persons were immediately isolated in their cabins and released 48 hours after symptoms resolved. On March 25, specimens were collected from 9 persons who remained in isolation; 7 (5 passengers and 2 crew members) tested positive for SARS-CoV-2 by reverse transcription PCR (RT-PCR). On the same day, 2 further persons evacuated for non–COVID-19 medical reasons also tested positive for SARS-CoV-2, and an outbreak was declared.

## Methods

### Command and Coordination

The government of Australia has legislated responsibility for human biosecurity for international maritime arrivals and took the lead role in a multiagency response to the outbreak. It tasked an Australian Medical Assistance Team (AUSMAT) to coordinate the operational aspects of managing the outbreak. The AUSMAT team worked closely with federal agencies involved in biosecurity and border control, the state health department and law enforcement agency, and the local port authority.

### Onboard Population Density Reduction

Vessel command divided the crew remaining on board into 2 groups, determined by the Minimum Safe Manning Certificate of the vessel: essential crew (EC), whose role was to maintain the safety (fire-fighting capacity, mooring lines) and vital functions (power supply and remote or direct systems monitoring) of the ship, and nonessential crew (nEC). Before quarantine began, all known SARS-CoV-2–positive case-patients (7 passengers and 2 crew members), along with their cabin-sharing contacts (7 passengers and 2 crew members) disembarked and were transferred to a hospital or hotel, depending on their clinical condition. An additional 2 passengers disembarked for other medical reasons and tested positive for SARS-CoV-2 upon hospital admission. Once they disembarked, no passengers or crew members returned to the ship, even if cleared of SARS-CoV-2 infection. Asymptomatic persons from Europe (791 passengers and 23 nEC) who were medically fit-to-fly (not tested for SARS-CoV-2 infection) repatriated to Germany on March 29, 2020, aboard 4 Condor Flugdienst charter flights.

### Case Identification and Management

We defined a case according to Australia’s public health guidelines for COVID-19 (*21*): a suspected case required symptoms of acute respiratory infection or a temperature of >38.0°C, and a confirmed case required a positive test result by RT-PCR on an oropharyngeal and bilateral deep nasal specimen. After commencement of quarantine, a health questionnaire based on the same guidelines was used for daily screening of nEC by using a cloud-based short message service (SMS) system or fixed telephone lines in cabins. Any health screening failure prompted a face-to-face interview and temperature measurement; otherwise, a face-to-face interview was conducted every 3 days. EC were monitored by daily face-to-face health screening and temperature measurement. The ship’s doctor (also in quarantine on board) provided additional information daily because crew reported symptoms directly. We collated and analyzed data pertaining to demographics, symptomatology, temperature recording, and laboratory results in Excel (Microsoft, https://www.microsoft.com).

### Laboratory Methods

Oropharyngeal and bilateral deep nasal swab samples were obtained from any crew member with symptoms, either self-reported or elicited during health screening, for SARS-CoV-2 testing. The swabs were placed in viral transport medium and stored at 4°C–8°C before testing. Testing was conducted at PathWest Laboratory Medicine WA by using a combined in-house RT-PCR directed at envelope and spike protein gene targets. This work was deemed a routine public health investigation and response, and no ethics approval was required.

### Operational Aspects of the Outbreak Response

#### Vessel Cleaning and Disinfection

**Figure 1:**
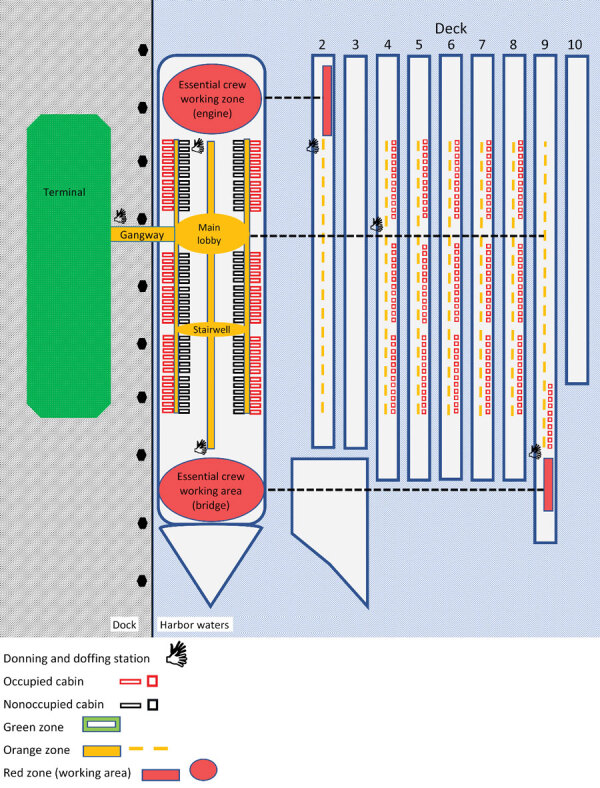
Layout of control zones for quarantine management aboard cruise ship used as quarantine facility to control onboard coronavirus disease outbreak, Western Australia, Australia. The terminal and dock were considered decontaminated (green zones); occupied cabins and work areas were considered contaminated (red zones); accessways from the shore to contaminated areas were considered buffer zones (yellow zones). Donning and doffing stations were placed at transition points between each zone.

Before quarantine began, a 30-person commercial cleaning team conducted a hospital-grade (*22*,*23*) environmental disinfection. The common areas of decks 4–9 were targeted first (Figure 1). The aim was to create decontaminated access areas that would be used by external medical, catering, and security personnel. Frequently touched surfaces and floors in common areas were cleaned daily. Cabins where the nEC were to be quarantined were decontaminated the next night, after which EC work areas were cleaned. EC work areas were cleaned to environmental standards but were considered contaminated because of ongoing work traffic from potentially infected EC during quarantine. Cleaning equipment was disinfected daily.

#### Crew Segregation

EC were accommodated in their own cabins and allowed to go to their designated work areas (bridge and engine room) and to respond to vessel emergencies. All nEC were accommodated individually in a separate area of the vessel in either unused or decontaminated cabins vacated by the disembarked passengers. They remained in strict quarantine for 14 days. EC and nEC could disembark, under escort, only if they tested positive for SARS-CoV-2, for other medical reasons, or because of a vessel emergency. The doors of all occupied cabins were marked to identify crew and food drop locations and for emergency evacuation purposes.

#### Infection Zones

The dock alongside the vessel and the adjacent terminal building were considered free of SARS-CoV-2 contamination (green zone). The access gangway, stairwells, and corridors to cabins were considered at low risk for contamination (yellow zone) and functioned as buffer zones. Contaminated areas (red zones) consisted of EC work areas and all occupied cabins (Figure 1).

#### Personal Protective Equipment Requirements

Entry to the green zone did not require personal protective equipment (PPE), but surgical masks and gloves were required for entry to the yellow zone. Within the red zone, different levels of PPE were mandated (Table). All external contractors were trained in PPE procedures, and AUSMAT monitored compliance at entry and exit points.

**Table:** PPE requirements in control zones on cruise ship used as a quarantine facility to control onboard COVID-19 outbreak, Western Australia, Australia*

Cohort	Location or activity	PPE requirement
All crew	Own cabin	Not required
	Own cabin balcony	Surgical mask
	Food collection and cabin waste removal	Surgical mask, gloves, distancing of 2 m
Essential crew	Routine duties in normal work zones	Surgical mask, gloves, and distancing of 2 m unless impossible because of the nature of the work carried out
	Emergency duties outside normal work zones	Tyvek suit, surgical mask, gloves, distancing of 2 m unless impossible because of the nature of the work carried out
Health team	Vessel-based telephone health screening	Surgical mask, gloves, distancing of 2 m
	Face-to-face cabin visit	N95 mask, protective eyewear, impervious gown and gloves
External contractors	CCTV monitoring desk, roving security, food delivery	Surgical mask, gloves, distancing of 2 m
	Waste removal	Impervious protective Level C suits, respirator masks, and protective eyewear

*CCTV, closed-circuit television; COVID-19, coronavirus disease; PPE, personal protective equipment.

#### Health and Well-being

Access to interpreter services was available, but because the official language on board was English, all crew had a reasonably good command of the language. All could communicate by using their own mobile phones (top-up credit vouchers were provided) and fixed-line telephones in cabins. AUSMAT also received, attended, and assessed health-related calls from crew. Initial contact was by telephone and escalated to a cabin visit or engagement of onshore WA health emergency resources, if required. Medical facilities on board were not used.

Efforts to minimize psychological stress and feelings of isolation and improve compliance included encouraging communication by individual 2-way and mass-SMS messaging systems, by using daily health checks as opportunities for high-quality contact time, and by using the public address and closed-circuit television (CCTV) systems to keep crew accurately informed. Other measures included acknowledging special events (birthdays and religious days), daily brain teaser exercises, and unsolicited local community support (handwritten postcards from primary school students).

#### Food Preparation, Supply, and Delivery

Before quarantine began, refrigerators in each cabin were stocked with several days’ supply of bottled water and long-life food and beverage items. To limit potential fomite spread, kitchen and catering facilities on board were not used. A 15-person external catering company prepared and delivered food for all persons on board, under direct supervision of AUSMAT. No food allergies were declared and meals consisted of culturally appropriate dish options not dissimilar to those normally available on board.

Food delivery was conducted through evening-only food-drops, consisting of a cold breakfast and lunch and hot dinner. Food was dropped in front of each cabin, and the bridge informed the relevant nEC by public address to open the door and collect the food package after delivery.

#### Waste Collection and Removal

Waste bags were prelocated in each cabin, collected from the front of each cabin, and disposed of by the nightly cleaning team. Judicious food packaging resulted in minimal waste.

#### Laundry and Linen

To minimize traffic, 2 sets of bed linens were placed in each occupied cabin; a contingency procedure for special circumstances was available through an external contractor. At the end of the quarantine period, all laundry and linen were collected in plastic bags and heat-cleaned at 60°C by using the washing facilities aboard the vessel.

#### Security

A comprehensive brief detailing the quarantine process, requirements, and restrictions was communicated to the crew. Compliance was continuously monitored by a temporary 16-camera internal CCTV system, supplemented by 5 security guards whose responsibilities included immediately, reporting any breaches of quarantine protocols.

## Results

### Description of the Outbreak

Before quarantine began on April 3, a total of 28 of 832 passengers and 30 of 503 crew members experienced symptoms and tested positive for SARS-CoV-2. The earliest symptoms in crew were recorded on March 21 in a motorman who later tested positive for SARS-CoV-2. We identified 2 distinct crew clusters: 5 security guards in whom symptom onset occurred during March 25–April 2, and 9 food service staff (6 wait staff and 3 food preparation staff) in whom symptom onset occurred during March 22–30.

During quarantine, 39 nEC disembarked: 21 (4.8%) symptomatic and SARS-CoV-2– positive persons (18 men and 3 women; mean age 41 years), and 18 close contacts, none of whom tested positive. After clearance testing on day 13 of quarantine, 2 asymptomatic EC tested positive, which resulted in all EC disembarking for a further 14-day onshore quarantine. All close contacts remained negative for SARS-CoV-2. A previously identified EC backup team from nEC subsequently managed the vessel.

**Figure 2:**
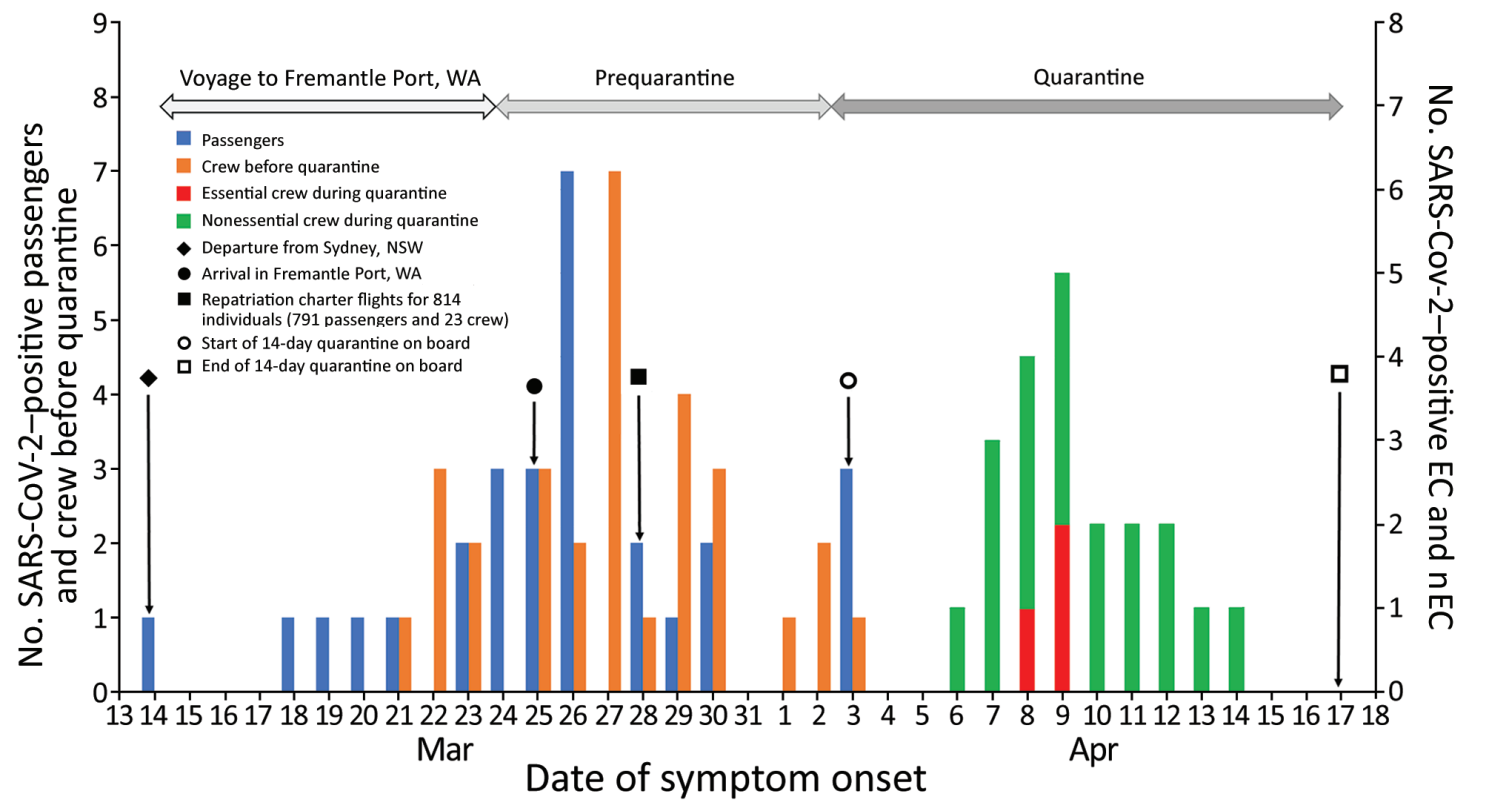
Epidemic curve of passenger and crew coronavirus disease cases by date of symptom onset aboard the MS Artania, WA, Australia, March 14–April 17, 2020. EC, essential crew; nEC, nonessential crew; NSW, New South Wales; SARS-CoV-2, severe acute respiratory syndrome coronavirus 2; WA, Western Australia.

**Figure 3:**
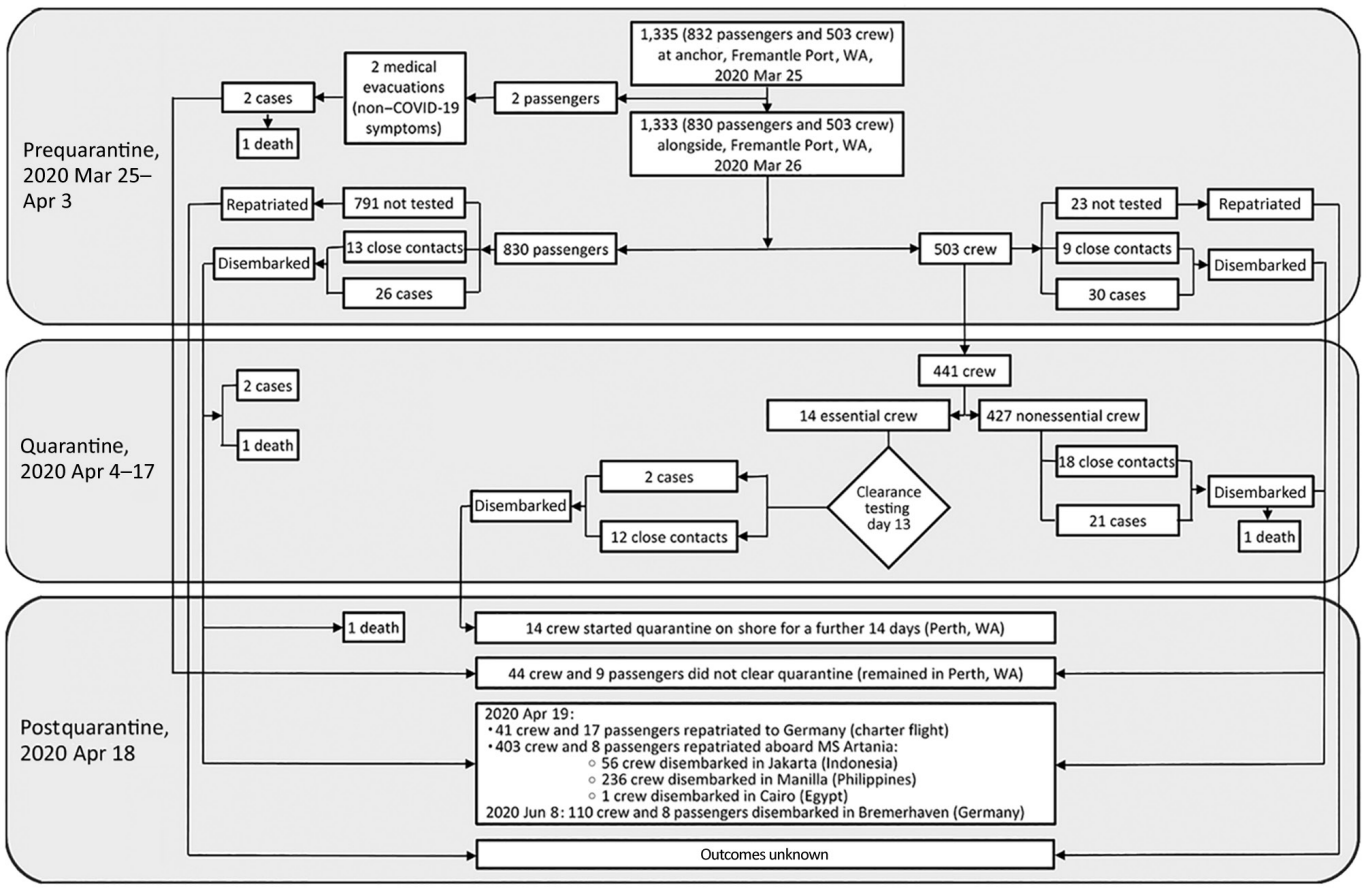
Flowchart of coronavirus disease outbreak outcomes of passengers and crew of the MS Artania, WA, Australia, March 14–April 18, 2020. WA, Western Australia.

The attack rate in crew before quarantine was 6.0% (30/503); during quarantine, the rate was 4.8% (21/441) (4.2% [18/427] in nEC and 21.4% [3/14] in EC). We recorded 1 COVID-19–related death in a male crew member 42 years of age. By the end of quarantine, 81 persons (30 passengers and 51 crew members) tested positive for SARS-CoV-2 (Figure 2); of those, 3 passengers and 1 crew member died (Figure 3). 

### Management of Health and Well-being Aspects during Quarantine

We performed health screening through 2,934 SMS messages, 3,339 telephone calls, and 1,033 face-to-face visits; we also reviewed 13 medical calls made by crew to the onboard doctor. A total of 245 RT-PCR tests were performed, including those used for clearance testing. No serious mental or physical health issues were reported; the main complaints conveyed by crew were constipation, lack of access to exercise, and lack of fresh air in some cabins.

A breach of quarantine was reported on day 5 when 3 nEC shared a kettle between adjacent rooms. Subsequently, 1 nEC became symptomatic and tested positive for SARS-CoV-2. A member of the external catering staff reported headache and fever (38°C) on day 7 of quarantine. She tested negative for SARS-CoV-2 and quarantined at home until symptoms resolved.

### Postquarantine Period

MS Artania departed Fremantle on April 18, 2020, carrying 403 crew and 8 passengers to return to its home port in Bremerhaven, Germany. During the voyage, crew were repatriated in Jakarta, Indonesia (56 crew members); Manilla, Philippines (236 crew members); and Cairo, Egypt (1 crew member). Because of the possibility of asymptomatic infection and transmission during the 51-day voyage, the vessel command continued to impose AUSMAT recommendations for social distancing and mask-wearing in communal areas. The medical team continued rigorous COVID-19 symptom screening and temperature measurement of all persons. No crew members demonstrated elevated temperature or symptoms of acute respiratory infection before arrival in Germany on June 8, 2020 (W. Roeske, MS Artania Medical Team, pers. comm., 2020 May 31).

## Discussion

Outbreaks of infectious diseases on cruise ships are a known risk, and cruise companies are well-versed in managing outbreaks of various types. However, SARS-CoV-2 poses a new and more severe threat, for which established prevention and response methods are inadequate. Early in the pandemic, several COVID-19 outbreaks on cruise ships drew global attention, and the level of risk and complexities involved in their control led to a shutdown of the cruise industry. We demonstrate that under certain circumstances, a COVID-19 outbreak aboard a cruise ship can be successfully controlled by using the vessel as a quarantine facility, which can have substantial financial, operational, and safety advantages.

The outbreak aboard the MS Artania occurred in a setting of low prevalence of the disease in the WA community but intense political and community concern about the risk of importing the virus into the state (*24*,*25*). Strong economic, political, and health and welfare imperatives existed to end the outbreak safely, effectively, and as quickly as possible, to enable the vessel to leave Australia’s waters and return to Germany. These conditions required that we establish minimum requirements to main the function and safety of the vessel while we enacted a stringent quarantine process using a holistic approach guided by established infection prevention and control (IPC) principles and in consideration of the welfare of all persons on board (*16*,*18*,*26*–*30*).

In many ways, a cruise ship is an appropriate environment to conduct a large-scale quarantine operation. Its many well-appointed accommodation spaces enable the isolation of a large number of persons comfortably and with good communication options. The main alternative—removing crew and passengers and housing them onshore—introduces sizeable cost and additional risk for infection transmission in the transfer process.

This outbreak resulted in 51 known cases and 1 death in crew members and 30 cases and 3 deaths in passengers. Isolation of case-patients, quarantining of exposed persons, and segregating onboard crew into EC and nEC groups were key response measures. To maximize the number of noninfected crew available to sail the vessel at the end of quarantine, and to reduce the quarantine duration, crew members were confined to their own cabins with nonshared facilities. EC, however, were required to perform their essential duties on board and thus were not in strict isolation. EC were not permitted to share food and were always requested to observe infection-prevention measures, but their entire working area could not be monitored by CCTV. The detection of 2 cases on day 13 might have been the result of a breach in infection-prevention measures during quarantine.

One key factor in determining the feasibility of using a ship as a quarantine facility is the number of cabins required to quarantine persons separately. In this outbreak, we achieved appropriate cabin numbers by disembarking passengers for repatriation or hospitalization before quarantine began, enabling the shortest possible time to prepare and conduct the quarantine: 20 days from the decision to quarantine to the ship’s departure from Fremantle Port. If there had been too few cabins to accommodate individual quarantine, regular RT-PCR testing of those who were sharing cabins would have enabled an early separation of discordant cabin mates, minimizing the overall period of quarantine.

Strict adherence to IPC was another tenet of our quarantine operation. The ship was separated into areas that reflected the level of risk for contamination and infection. Thorough daily cleaning maintained the status of these zones, and PPE requirements for each zone were rigidly enforced. Strict control of food preparation and delivery was a key component of the quarantine process. The use of external caterers mitigated the risk for fomite transmission through food prepared by potentially infected crew. The food-drop system essentially eliminated direct contact between catering staff and crew, negating the need for high-level PPE.

The presence of roving security personnel and the installation of CCTV cameras to monitor adherence to quarantine proved useful in 2 ways. These measures acted as incentives for quarantined crew to remain secluded in their rooms and ensured that any breaches were recognized and infection risk managed. The swift alert to the quarantine breach among 3 nEC enabled immediate review of events and decisive action.

The low level of SARS-CoV-2 activity in the WA community at the time of the operation, coupled with temperature and symptom screening of all responders (AUSMAT and contractors), gave us a high level of confidence that the WA responders were not themselves a risk vector for infection. In geographic locations where SARS-CoV-2 activity is higher, introduction of the virus on board by infected responders would need to be mitigated by regular symptom and temperature checks, SARS-CoV-2 testing, or both.

Solitary quarantine is challenging and potentially detrimental to physical and mental health. We focused on minimizing the length of quarantine through strict adherence to its principles and excellent communication by using a variety of technologies. Daily health checks provided the opportunity to build rapport and support and to reinforce and encourage compliance with quarantine requirements, and efforts were made to acknowledge special events. This approach might have contributed to a lack of reported serious mental or physical issues. The ultimate measure of success of this operation was that no symptoms consistent with SARS-CoV-2 infection were detected in any crew member after the 14-day quarantine period on board ended.

The first limitation of our study is that it does not provide a complete description of the outbreak on board the MS Artania. Whereas no passengers or crew had to quarantine or be tested upon arrival in Bremerhaven, some crew who disembarked in other countries had to quarantine or be tested upon arrival home. None of these crew were symptomatic, but we could not obtain further details after their arrival. Our attempts to identify other cases from the cohort of repatriated passengers and crew before the start of quarantine have not been successful. Second, we could not be certain that all asymptomatic nEC were not infected and infectious during quarantine and at its conclusion because we did not test asymptomatic nEC as a condition of release. However, none subsequently experienced symptoms of COVID-19, and ongoing IPC measures for the duration of the voyage back to Germany mitigated this small risk even further. The guidelines for screening and testing for SARS-CoV-2 are constantly evolving. Our screening and testing protocols reflected best practices in Australia at that time, and a similar vessel outbreak would now be managed under a more rigorous testing regime.

Although the international cruise industry was effectively halted because of the COVID-19 pandemic, some cruises have restarted and the risk for COVID-19 outbreaks will endure. The severe consequences of such outbreaks to human life and to the viability of the cruise industry necessitate a precautious approach, including the ability to manage outbreaks effectively and efficiently.

In conclusion, use of the ship itself as a quarantine facility during an onboard outbreak offers financial and operational advantages, and we have demonstrated its feasibility under certain circumstances. Onboard quarantine should be considered as an option in COVID-19 outbreak response plans for cruise ships.

## References

[1] Isakbaeva ET, Widdowson M-A, Beard RS, Bulens SN, Mullins J, Monroe SS, et al. Norovirus transmission on cruise ship. Emerg Infect Dis. 2005;11:154–8. 10.3201/eid1101.040434 15705344 PMC3294347

[2] Brotherton JML, Delpech VC, Gilbert GL, Hatzi S, Paraskevopoulos PD, McAnulty JM; Cruise Ship Outbreak Investigation Team. A large outbreak of influenza A and B on a cruise ship causing widespread morbidity. Epidemiol Infect. 2003;130:263–71. 10.1017/S0950268802008166 12729195 PMC2869962

[3] Mouchtouri VA, Rudge JW. Legionnaires’ disease in hotels and passenger ships: a systematic review of evidence, sources, and contributing factors. J Travel Med. 2015;22:325–37. 10.1111/jtm.12225 26220258

[4] Minooee A, Rickman LS. Infectious diseases on cruise ships. Clin Infect Dis. 1999;29:737–43, quiz 744. 10.1086/520426 10589880

[5] Rooney RM, Cramer EH, Mantha S, Nichols G, Bartram JK, Farber JM, et al. A review of outbreaks of foodborne disease associated with passenger ships: evidence for risk management. Public Health Rep. 2004;119:427–34. 10.1016/j.phr.2004.05.007 15219800 PMC1497653

[6] Lanini S, Capobianchi MR, Puro V, Filia A, Del Manso M, Karki T, et al. Central task force for measles outbreak. Measles outbreak on a cruise ship in the western Mediterranean, February 2014, preliminary report. Euro Surveill. 2014;19:10. 10.2807/1560-7917.ES2014.19.10.20735 24650863

[7] World Health Organization. Considerations for quarantine of contacts of COVID-19 cases, 2020 [cited 2020 Mar 26]. https://www.who.int/publications/i/item/considerations-for-quarantine-of-individuals-in-the-context-of-containment-for-coronavirus-disease-(covid-19)

[8] World Health Organization. Operational considerations for managing COVID-19 cases/outbreak on board ships. 2020 [cited 2020 May 1]. https://www.who.int/publications-detail/operational-considerations-for-managing-covid-19-cases-outbreak-on-board-ships

[9] Sehdev PS. The origin of quarantine. Clin Infect Dis. 2002;35:1071–2. 10.1086/344062 12398064

[10] Chimonas MA, Vaughan GH, Andre Z, Ames JT, Tarling GA, Beard S, et al. Passenger behaviors associated with norovirus infection on board a cruise ship—Alaska, May to June 2004. J Travel Med. 2008;15:177–83. 10.1111/j.1708-8305.2008.00200.x 18494695

[11] Millman AJ, Kornylo Duong K, Lafond K, Green NM, Lippold SA, Jhung MA. Influenza outbreaks among passengers and crew on two cruise ships: A recent account of preparedness and response to an ever-present challenge. J Travel Med. 2015;22:306–11. 10.1111/jtm.12215 26031322 PMC4869710

[12] Ward KA, Armstrong P, McAnulty JM, Iwasenko JM, Dwyer DE. Outbreaks of pandemic (H1N1) 2009 and seasonal influenza A (H3N2) on cruise ship. Emerg Infect Dis. 2010;16:1731–7. 10.3201/eid1611.100477 21029531 PMC3294517

[13] World Health Organization. WHO announces COVID-19 outbreak a pandemic. 2020 [cited 2020 Apr 12]. https://www.euro.who.int/en/health-topics/health-emergencies/coronavirus-covid-19/news/news/2020/3/who-announces-covid-19-outbreak-a-pandemic

[14] Moriarty LF, Plucinski MM, Marston BJ, Kurbatova EV, Knust B, Murray EL, et al.; CDC Cruise Ship Response Team; California Department of Public Health COVID-19 Team; Solano County COVID-19 Team. California Department of Public Health COVID-19 Team; Solano County COVID-19 Team. Public health responses to COVID-19 outbreaks on cruise ships—worldwide, February–March 2020. MMWR Morb Mortal Wkly Rep. 2020;69:347–52. 10.15585/mmwr.mm6912e3 32214086 PMC7725517

[15] Mizumoto K, Chowell G. Transmission potential of the novel coronavirus (COVID-19) onboard the diamond Princess Cruises Ship, 2020. Infect Dis Model. 2020;5:264–70. 10.1016/j.idm.2020.02.003 32190785 PMC7068636

[16] Rocklöv J, Sjödin H, Wilder-Smith A. COVID-19 outbreak on the Diamond Princess cruise ship: estimating the epidemic potential and effectiveness of public health countermeasures. J Travel Med. 2020;27:taaa030. 10.1093/jtm/taaa030 PMC7107563 32109273

[17] Kakimoto K, Kamiya H, Yamagishi T, Matsui T, Suzuki M, Wakita T. Initial investigation of transmission of COVID-19 among crew members during quarantine of a cruise ship—Yokohama, Japan, February 2020. MMWR Morb Mortal Wkly Rep. 2020;69:312–3. 10.15585/mmwr.mm6911e2 32191689 PMC7739985

[18] Dahl E. Coronavirus (Covid-19) outbreak on the cruise ship Diamond Princess. Int Marit Health. 2020;71:5–8. 10.5603/MH.2020.0003 32212140

[19] Japanese National Institute of Infectious Disease. Field briefing: Diamond Princess COVID-19 cases. 2020 Feb 20 [cited 2020 Mar 1]. https://www.niid.go.jp/niid/en/2019-ncov-e/9407-covid-dp-fe-01.html

[20] Centers for Disease Control and Prevention. Coronavirus disease 2019. 2020 [cited 2020 Mar 2]. https://www.cdc.gov/coronavirus/2019-ncov/index.html

[21] Communicable Diseases Network Australia. Coronavirus Disease 2019 (COVID-19): CDNA National Guidelines for Public Health Units [cited 2020 Mar 24]. https://www1.health.gov.au/internet/main/publishing.nsf/Content/cdna-song-novel-coronavirus.htm

[22] Australian Government Department of Health. Public Summary: Diversey Australia Pty Ltd—Oxivir FIVE16—disinfectant, hospital grade. 2017 [cited 2020 Apr 24]. http://www.diverseyvericlean.com/images/DiverseyHumming/TGA/PublicSummary/Oxivir-Five-16-JF-SD-3.78L---AUST-R-286618---Public-Summary.pdf

[23] Australian Government Department of Health. Coronavirus disease (COVID-19): environmental cleaning and disinfection principles for COVID-19. 2020 [cited 2020 May 13]. https://www.health.gov.au/sites/default/files/documents/2020/03/environmental-cleaning-and-disinfection-principles-for-covid-19.pdf

[24] Hedley KWA. Premier blindsided by news sick passengers still on cruise ship refusing to leave Freo. WA Today. 2020 Apr 1 [cited 2020 Apr 23]. https://www.watoday.com.au/national/western-australia/wa-premier-blindsided-by-news-sick-passengers-still-on-cruise-ship-refusing-to-leave-freo-20200401-p54g2k.html

[25] Western Australia Government. Emergency management act 2005 (WA) section 67: MS Artania Directions. 2020 [cited 2020 Apr 23]. https://www.wa.gov.au/sites/default/files/2020-03/Ms%20Artania%20Directions_0.pdf

[26] Boyce JM. Modern technologies for improving cleaning and disinfection of environmental surfaces in hospitals. Antimicrob Resist Infect Control. 2016;5:10. 10.1186/s13756-016-0111-x 27069623 PMC4827199

[27] Rutala WA, Weber DJ. Disinfectants used for environmental disinfection and new room decontamination technology. Am J Infect Control. 2013;41(Suppl):S36–41. 10.1016/j.ajic.2012.11.006 23622746

[28] Dancer SJ. Controlling hospital-acquired infection: focus on the role of the environment and new technologies for decontamination. Clin Microbiol Rev. 2014;27:665–90. 10.1128/CMR.00020-14 25278571 PMC4187643

[29] van Doremalen N, Bushmaker T, Morris DH, Holbrook MG, Gamble A, Williamson BN, et al. Aerosol and surface stability of SARS-CoV-2 as compared with SARS-CoV-1. N Engl J Med. 2020;382:1564–7. 10.1056/NEJMc2004973 32182409 PMC7121658

[30] Triandis CH. Interpersonal behaviour. Monterey (CA): Brooks/Cole Publishing Company; 1977.

